# Antler stem cell-derived exosomes promote regenerative wound healing via fibroblast-to-myofibroblast transition inhibition

**DOI:** 10.1186/s13036-023-00386-0

**Published:** 2023-11-08

**Authors:** Guokun Zhang, Dongxu Wang, Jing Ren, Jiping Li, Qianqian Guo, Liyan Shi, Chunyi Li

**Affiliations:** 1 Institute of Antler Science and Product Technology, Changchun Sci-Tech University, No. 1345 of Pudong Rd., Changchun, Jilin 130600 China; 2 College of Chinese Medicinal Materials, Jilin Agricultural University, Changchun, 130118 China; 3 China-Japan Union Hospital, Jilin University, Changchun, 130033 China

**Keywords:** Antler stem cells, Exosomes, Fibroblast-to-myofibroblast transition, Regenerative wound healing, Scarring

## Abstract

**Introduction:**

The typical outcome of mammalian wound healing is scarring, a fibrotic process mediated by myofibroblast aggregation. Perfect healing in a clinical setting is relatively unexplored. Surprisingly, our previous studies have shown that the large wound (10 cm diameter or more) of the pedicle of deer naturally achieves regenerative restoration, realized through a paracrine pathway from adjacent antler stem cells (AnSCs).

**Methods:**

AnSC-derived exosomes (AnSC-exos) were topically injected around the full-thickness wounds in a rat model. The effects on the rate of wound healing and the quality of healing were evaluated via morphological, histological, and molecular biological techniques on days 14 and 28 after surgery.

**Results:**

The results showed that AnSC-exos significantly accelerated the rate of wound healing and improved healing quality, including regeneration of cutaneous appendages (hair follicles and sebaceous glands) and the distribution pattern of collagen (basket-weave-like) in the healed skin. These effects of AnSC-exos were comparable to those of AnSCs but were significantly more potent than those of exosomes derived from bone marrow mesenchymal stem cells (bMSC-exos). Furthermore, AnSC-exos treatment effectively inhibited fibroblast-to-myofibroblast transition (FMT), as evidenced by the reduction of full-thickness skin injury-induced FMT in vivo and TGF-β1-induced FMT in vitro.

**Conclusion:**

AnSC-exos could effectively promote regenerative cutaneous wound healing, highly likely through FMT inhibition. This suggests that AnSC-exos treatment could provide the potential for a novel approach to induce regenerative wound healing in the clinical setting.

**Graphical Abstract:**

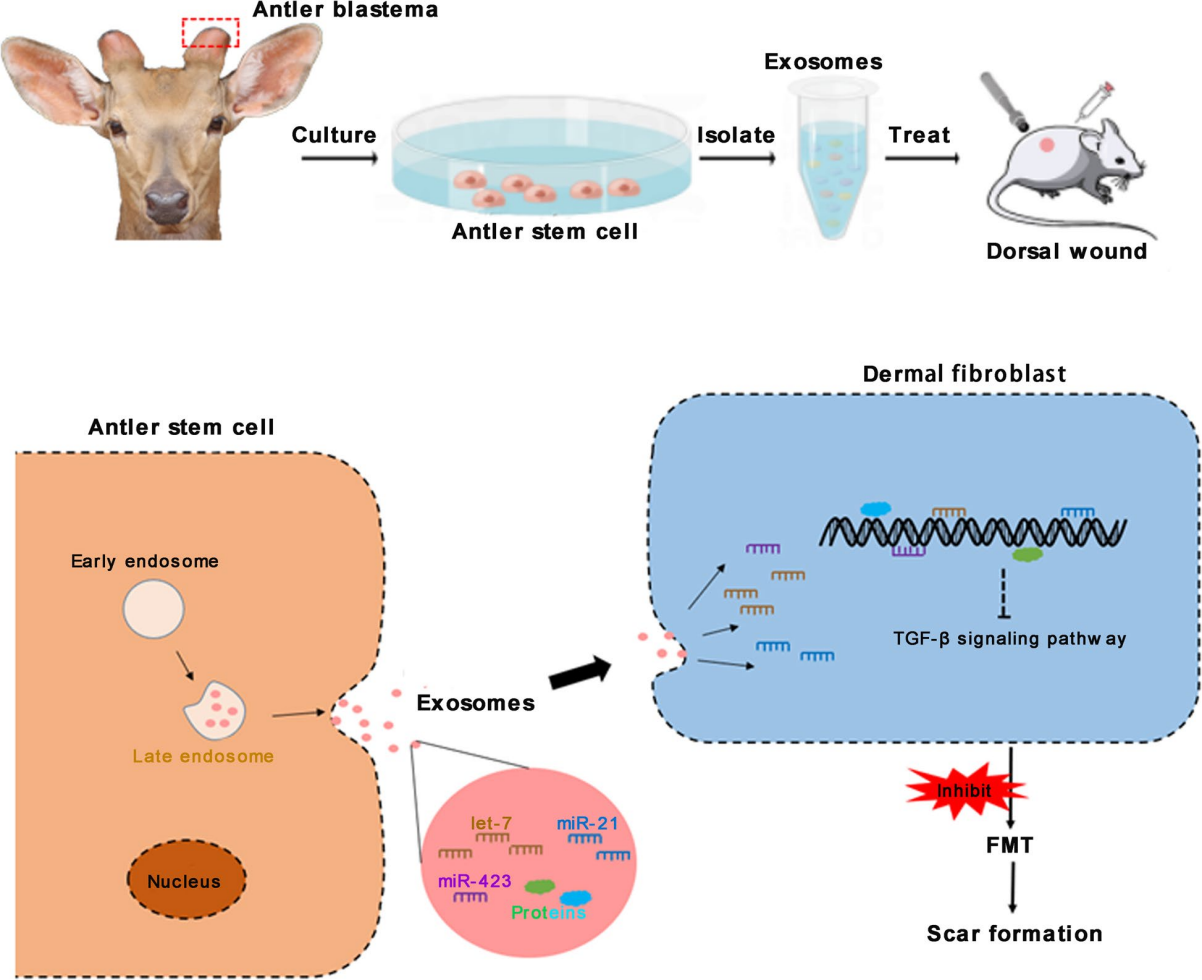

**Supplementary Information:**

The online version contains supplementary material available at 10.1186/s13036-023-00386-0.

## Highlights

• A novel type of stem cells, AnSCs, was found to be able to effectively promote regenerative cutaneous wound healing without scarring;

• The cell-free therapy, AnSC-exos administration, had comparable effects to AnSCs but more potent than bone marrow mesenchymal stem cell-derived exosomes on wound healing;

• AnSC-exos could effectively inhibit the fibroblast-to-myofibroblast transition (FMT) process.

**Supplementary Information:**

The online version contains supplementary material available at 10.1186/s13036-023-00386-0.

## Introduction

A fibrotic scar is the end point of wound healing in adult mammals [1, 2]. Unlike normal skin, the scar is rich in extracellular matrix (ECM), particularly collagen, and lacks cutaneous appendages [3–5]. Scars causes physical and psychological distress, and considerable medical and economic burdens [6, 7]. According to statistics, over 100 million scars are resulted from wound healing each year in the US, and cost the US over $20 billion [3, 8]. Hence, scar-free wound regeneration is the ultimate clinical goal [7], but despite decades of research, no therapy is currently available than can effectively preventing or reversing scarring. Therefore, superior strategies for reducing scars are urgently needed to be developed.

Fibroblasts are the key cell type that is involved in wound healing and appear in the healing wound 2 to 5 days after injury. These cells then proliferate, migrate and differentiate in actively participating wound healing [3, 8, 9]. Among them, myofibroblasts (as the predominant collagen-secreting cells) are one of the most critical contributors to scarring as they cause contraction of the wound and form thick parallel fiber bundles [10, 11]. Such scarring also affects the regeneration of cutaneous appendages. The major source of myofibroblasts comes from transdifferentiation of fibroblasts, a process termed fibroblast-to-myofibroblast transition (FMT) [9, 12]. Therefore, interference of FMT may provide an effective strategy to reduce scarring and achieve superior regenerative wound healing. Mesenchymal stem cell (MSC) therapy has been shown to be able inhibit FMT effectively and is a potential approach to promoting regenerative wound healing [13–15]. The advantages of MSC application include relatively easy expansion in vitro, the ability to homing in the injury site and the ability of the MSC to differentiate into specific cell types required for tissue repair/regeneration [14, 16–18]. However, the limited availability of somatic adult MSCs and embryonic stem cells (ESCs) hinder their application in the field of stem cell therapy [19].

Deer antler is the only mammalian organ that can fully regenerate from the permanent bony protuberances, known as pedicles [20–22]. Full antler regeneration initiates from the essentially scarless wound healing over the apex of a pedicle stump [23–26]. Studies have shown that this scarless wound healing depends on the adjacent antler stem cells (AnSCs) resident in the pedicle periosteum or in the early antler blastema [22, 25, 26]. Our studies have shown that, although AnSCs belong to a type of adult MSC, they express some critical ESC markers along with MSC markers, and possess potent proliferation and differentiation capacities [20, 27, 28]. Recently, we applied AnSCs to treat cutaneous wounds in rats and found AnSCs significantly accelerated the rate of healing and improved healing quality (restoring the cutaneous appendages and normal dermal structure) in a burn model [17]. Further study revealed that an AnSC conditioned medium (AnSC-CM) could largely replicate the effects of AnSCs on wound healing in rats [29], suggesting that a paracrine mechanism is likely responsible for the induction of regenerative wound healing by AnSCs. However, the specific paracrine components that promote wound regeneration are not known.

Exosomes are small vesicles secreted by cells with a diameter of about 30–150 nm, which mediate intercellular communication through mRNA, miRNA, DNA and proteins inside. Recently, exosomes have been recognized as the main actor in the therapeutic function of stem cells [30, 31]. This study aimed to examine the effects (in rats) of AnSC-derived exosomes (AnSC-exos) on regenerative wound healing including regeneration of cutaneous appendages and restoration of normal ECM structure. We found that AnSC-exos had comparable effects to AnSCs on wound healing, with both enhancing the rate of healing and improving the quality of full-thickness skin wounds. We also found that AnSCs may play this role via inhibiting FMT. Overall, the present study proposes a promising strategy to improve the quality of wound healing and opens up the potential opportunity for the application of AnSC-exos in the clinical setting to achieve scarless healing.

## Materials and methods

### Cell culture and characterization

AnSCs were isolated from the tissue of antler blastema (3–7 days after hard antler button casting; Jilin Dongao Deer Industry Group Co., Ltd.) obtained from a healthy 2-year-old male sika deer [22]. Skin covering the antler blastema was cut open to expose the blastema tissue, which was then removed and cut into 0.2 mm in cubes, washed three times with PBS, and digested in type I collagenase for 30 min (37 °C). The digested tissue was transferred to a 10 cm culture dish for primary cell culture. The cells were sub-cultured when they reached confluence and used in subsequent experiments (all within 5 passages).

AnSCs were characterized through profiling of marker gene expression (CD34, CD45, CD73, CD90, CD105, and SOX2) using both immunofluorescence (IF) staining and flow cytometry (FCM) as previously described [14, 27, 32]. Cultured AnSCs (≈ 70% confluence) were fixed with methanol and incubated with primary antibodies as noted. Procedures for all primary antibodies were essentially the same, except for SOX2 in that staining needs to be preceded by Triton X-100 for 10 min. Secondary antibodies were incubated, followed by nuclear staining with DAPI (Beyotime, China) and photography with a fluorescence microscope (VOS M5000, USA). The cell suspensions were sequentially stained with AF488-labeled secondary antibodies and then quantitatively analyzed using BD FACSCelesta (BD Biosciences, USA). The antibodies are listed in Table S1.

Rat bone marrow MSCs (bMSCs) and human dermal fibroblasts (hDFs) were cryopreserved in our laboratory. The cells, including AnSCs, bMSCs and hDFs, were all cultured in DMEM (Gibco, USA) containing 10% fetal bovine serum (FBS; Gibco, USA) and supplemented with 1% penicillin/streptomycin (BI, Israel) at 37 °C, 5% CO_2_, saturated humidity.

### Exosome preparation

AnSCs were cultured in the FBS-containing DMEM medium, and when they reached around 90% confluence, the medium was replaced with a serum-free medium (Hyclone, USA) for 48 h. The conditioned medium was subsequently collected, filtered through a 0.1-µm filter device and ultra-centrifuged at 100,000 g for 3 h to isolate exosomes [33, 34]. The exosomes were diluted with PBS and stored at -80℃ for later use. Confirmation of AnSC-exos was performed using transmission electron microscopy, exosomal marker (CD9, CD63, and TSG101) detection, and NanoSight NS300 (Malvern Instruments, UK) [30].

### Wounding experiment

Wounding experiments were carried out on 64 rats (female, eight-week-old) under full anesthesia. All animal experiments were approved by the Animal Ethics Committee of Changchun Sci-Tech University (No. CKARI202005). The rats were anesthetized with 3% pentobarbital sodium (30 mg/kg), the dorsal hair was shaved, and then the full-thickness skin with a diameter of 12 mm along the center of the back was excised without damaging the subcutaneous muscle tissue using a skin biopsy punch [29, 35]. Rats were randomly divided into four groups (16 rats/group): AnSCs (2 × 10^6^ cells), bMSC-exos (50 µg), AnSC-exos (50 µg), and control (PBS). Weekly local application of cells or exosomes through injection around the wound margins (25 µl per injection × 4 injections), respectively. The wounds were photographed at four-day intervals, and the wound area (area not covered by the epidermis where the scab is included in the wound) at each time point was calculated using Image J software. Half of the rats were euthanized on 14 or 28 postoperative days (POD14 or POD28), and healing/healed skin samples were collected for histological examination and molecular analyses.

### FMT induction and AnSC-exos treatment (in vitro)

The hDFs were seeded into a 24-well plate at a density of 50,000 cells/well (500 µl). There were four treatment groups (3 wells/treatment): (1) general control (intact), (2) PBS, (3) bMSC-exos, and (4) AnSC-exos. After 12 h culture, TGF-β1 (25 ng/ml/well) was added together with either PBS (20 µl), bMSC-exos (100 ng/ml/well, 20 µl) or AnSC-exos (100 ng/ml/well, 20 µl) for further 48 h to the treatment groups (2, 3, 4) to induce FMT. Expression levels of α-Smooth muscle actin (α-SMA) were measured using IF staining and western blot analysis.

### Histology

Sampled wound tissues were fixed in 4% paraformaldehyde for two days and rinsed with water. Following sequential dehydration in gradient ethanol and xylene, tissues were dehydrated and embedded in paraffin wax, and cut at 5.0 μm thickness. The sections were stained with hematoxylin-eosin (HE) and Masson following the manufacturer’s instructions. The numbers of cutaneous appendages per 20×high-power field (HPF) were counted manually. The total amount of collagen per HPF was quantified using the image of Masson staining per Image-Pro Plus software.

Cytokeratin 14 (CK14), Cytokeratin 19 (CK19), α-SMA and Transglin (TAGLN) were used for IF staining of paraffin sections. Secondary antibodies were incubated, followed by nuclear staining with DAPI and photography with a fluorescence microscope. The number of cells per HPF that were showing positive expression was counted manually. The antibodies are listed in Table S1.

### RNA analyses

Total RNA of each tissue type was isolated using Trizol (Invitrogen, USA) and the cDNA was generated with a cDNA Synthesis Kit (Takara, Japan) from RNA. The qRT-PCR was performed to determine the expression levels of target RNAs using SYBR Green Master (Roche, Switzerland) in qTOWER 3G (Analykit Jena AG, Germany). The mRNAs were quantified using ^ΔΔ^Ct method. The primers are listed in Table S2.

### Western blot

Total proteins were isolated from the tissue samples and hDFs. 20 µg of total protein was separated via polyacrylamide SDS gel and then transferred to a polyvinylidene fluoride membrane (Millipore, MA). After blocking in 5% (w/v) non-fat milk, the membrane was first incubated with primary antibodies at 4℃ overnight and then with HRP-conjugated secondary antibody at 25℃ for 2 h. The primary antibodies are listed in Table S1. The bands were visualized using an ECL system and quantified using Image J software.

### Statistical analysis

Data were presented as mean ± SD. Statistical analysis was performed by one-way ANOVA using GraphPad Prism software. *P* < 0.05 was considered statistically significant.

## Results

### Characterization of AnSCs and preparation of AnSC-exos

**Fig. 1:**
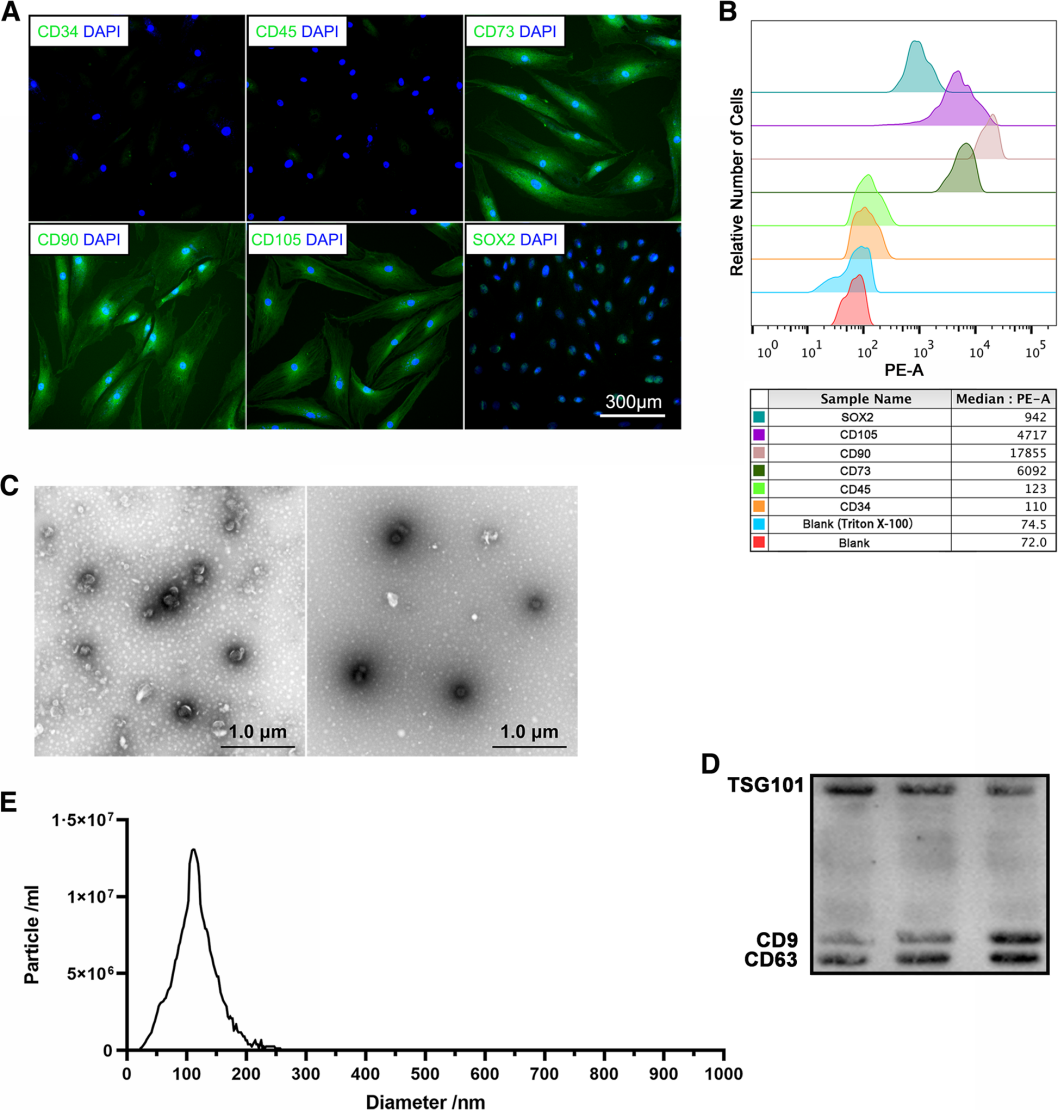
Characterization of AnSCs and preparation of AnSC-exos. **A**, **B** The cells isolated from the initial antler blastema were identified via flow cytometry and immunofluorescence staining. **C** Morphology of AnSC-exos via transmission electron microscopy. **D** Detection of exosomal markers in AnSC-exos via western blot. **E** Particle size of AnSC-Exos via NanoSight. AnSCs, antler stem cells; AnSC-exos, AnSC-derived exosomes

AnSCs were identified via IF staining and FCM analysis: AnSCs were positive for CD73, CD90, CD105 and Sox2 and negative for CD34 and CD45 (Fig. 1A, B). Results showed that AnSC-exos were roundish (Fig. 1C) with an average size of 120 nm in diameter (Fig. 1E), and expressed exosomal markers (CD9 and CD63, and TSG101) (Fig. 1D).

### AnSC-exos accelerated healing rate in rat full-thickness wounds

**Fig. 2:**
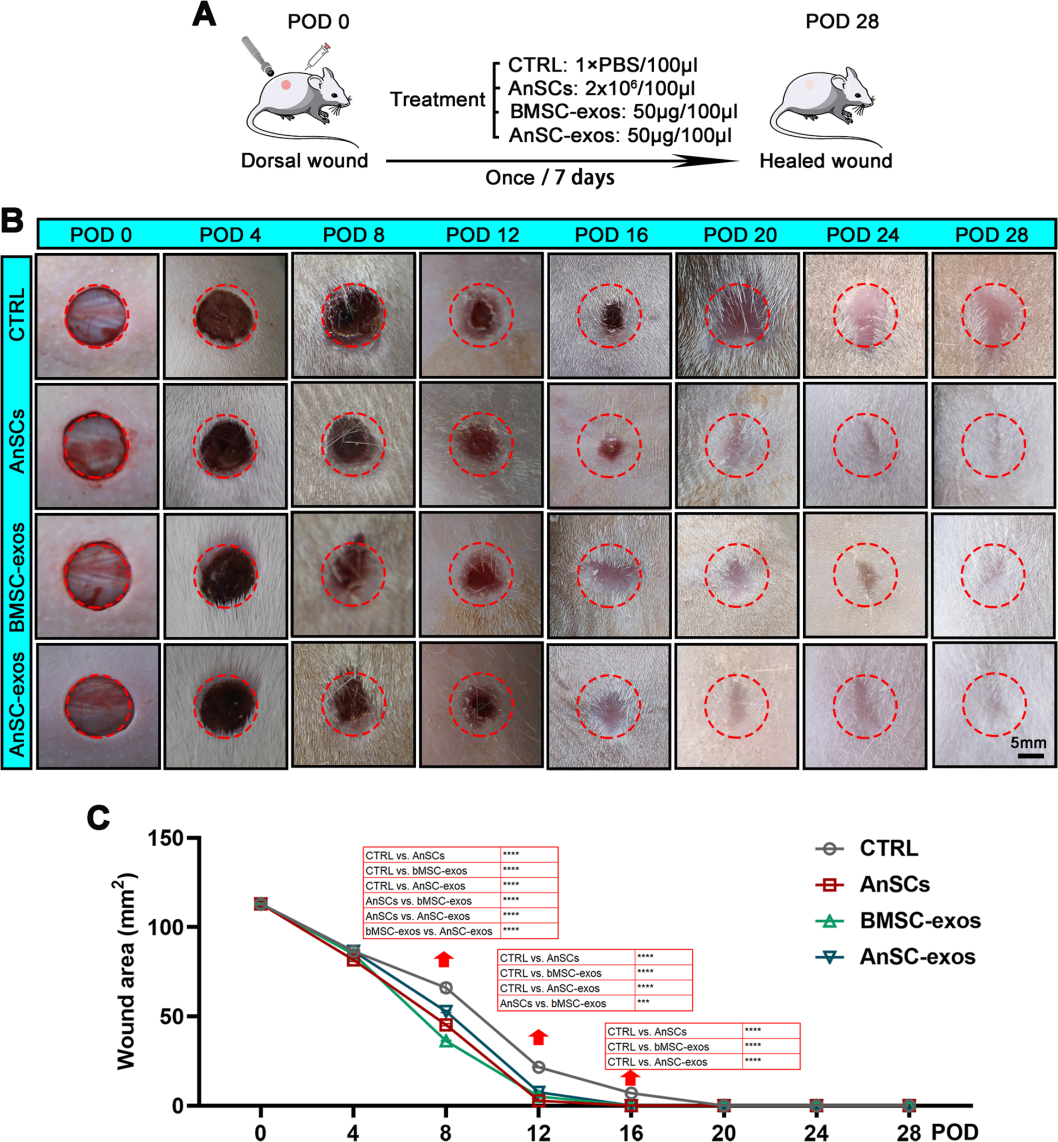
Effects of AnSC-exos on rate of wound healing in rats. **A** Schematic diagram of the experimental design. **B** Gross photographs of rat dorsal full-thickness wounds during the healing period. **C** Quantitative evaluation of wound area during healing. Note: AnSC-exos promoted wound healing rate and reduced scarring, and these effects were comparable to AnSCs, but more potent than bMSC-exos. Mean ± SEM; ***P < 0.001, ****P < 0.0001, *n* = 8. bMSC-exos, bone marrow mesenchymal stem cell-derived exosomes; CTRL, control; POD, postoperation day

Full-thickness wounds were created in rats to evaluate the effects of AnSC-exos on the rate of healing; PBS was used as a negative control, AnSCs and bMSC-exos were used as the positive controls (Fig. 2A). Results showed that on POD8 and POD12, significant differences in wound areas (mm^2^) were detected between the different groups: POD8 - CTRL (66.0 ± 3.61) > AnSC-exos (52.8 ± 0.86) > AnSCs (45.4 ± 0.74) > bMSC-exos (36.3 ± 0.59); POD 12 - CTRL (21.6 ± 0.35) > bMSC-exos (7.5 ± 0.86) > AnSC-exos (5.2 ± 0.17) > AnSCs (2.8 ± 0.43) (Fig. 2B and C). On POD16, the wounds in the AnSCs, bMSC-exos, and AnSC-exos groups were essentially closed; whereas, in the CTRL group, the wounds were still evident (7.1 ± 0.12) and closed on POD20 (Fig. 2B and C).

### AnSC-exos promoted cutaneous appendage regeneration

**Fig. 3:**
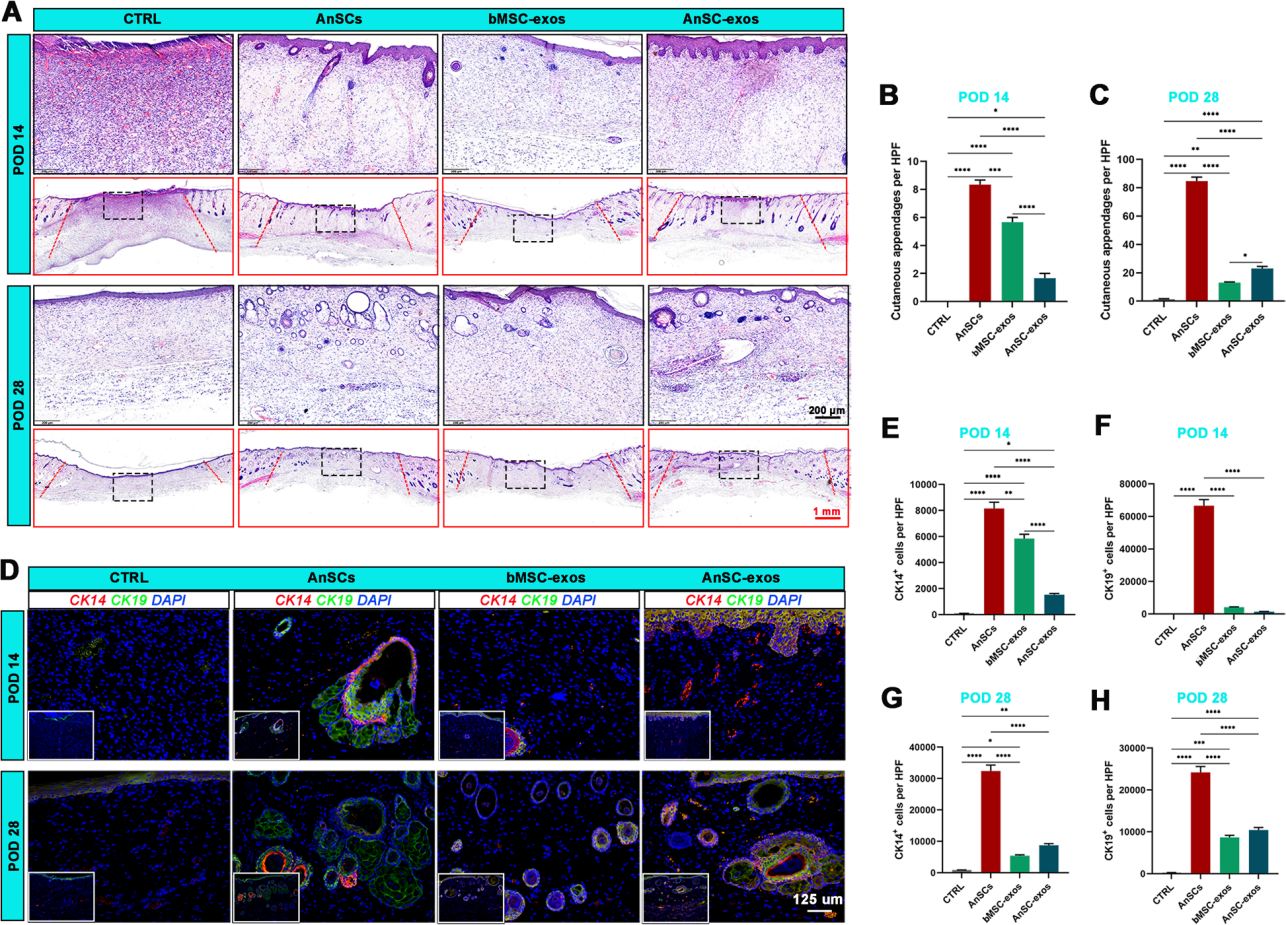
Effects of AnSC-exos on regeneration of cutaneous appendages in rats. **A** Histological sections of the healed skin stained with HE. **B**, **C** Number of cutaneous appendages in the healed skin on POD 14 and POD 28. **D** CK14 and CK19 immunofluorescence staining. **E**, **F** Number of CK14^+^ cells and CK19^+^ cells on POD 14. **G**, **H** Number of CK14^+^ cells and CK19^+^ cells on POD 28. Note: AnSC-exos promoted cutaneous appendage regeneration, and the overall effects were comparable to those of AnSCs, but more potent than that of bMSC-exos. Mean ± SEM; *P < 0.05, **P < 0.01, ***P < 0.001, ****P < 0.0001; *n* = 3. CK14, cytokeratin 14; CK19, cytokeratin 19; HPF, 20× high-power field

The quality of wound healing was examined at the histological level. On POD14, re-epithelialization in the CTRL group was incomplete with the wound surface filled with numerous monocytes-macrophages; in contrast, in the AnSCs, bMSC-exos and AnSC-exos groups were complete. On POD28, re-epithelialization of the wounds in the CTRL was complete. The epidermis, dermis and subcutaneous loose connective tissue in each group were clearly delineated (Fig. 3A). The number of cutaneous appendages (hair follicles and sebaceous glands) in the healed skin was counted on the HE-stained image. There was no evidence of regenerated cutaneous appendage in the CTRL group on POD14 or POD28. In contrast, regenerated cutaneous appendages were evident in the healed tissue of the AnSCs, bMSC-exos and AnSC-exos groups, although the number of regenerated appendages in the AnSC-exos group was less than in the other two groups (Fig. 3A, B; *P *< 0.0001) on POD14. On POD28, the number of regenerated cutaneous appendages in the healed skin showed a similar trend, but there were significantly more in the AnSC-exos group than in the bMSC-exos group (Fig. 3A, C; *P* < 0.05).

Next, IF staining was performed to visualize expression of specific markers CK14 and CK19 in hair follicles and sebaceous glands. There was no evidence of CK14^+^ cells and CK19^+^ cells in the CTRL group on POD14 or POD28 in the healed tissue; whereas, numerous CK14^+^ cells and CK19^+^ cells were detected in the AnSC-exos group, although there were fewer in this group compared with the AnSC group on POD14 (Fig. 3D-F; *P* < 0.0001) and on POD28 (Fig. 3D, G, H; *P* < 0.0001). Overall, the results suggest that treatment with AnSC-exos can effectively promote regeneration of cutaneous appendages in the healing tissue in rats, and that the effects are comparable to those of the AnSCs.

### AnSC-exos improved the structure and type of collagens

**Fig. 4:**
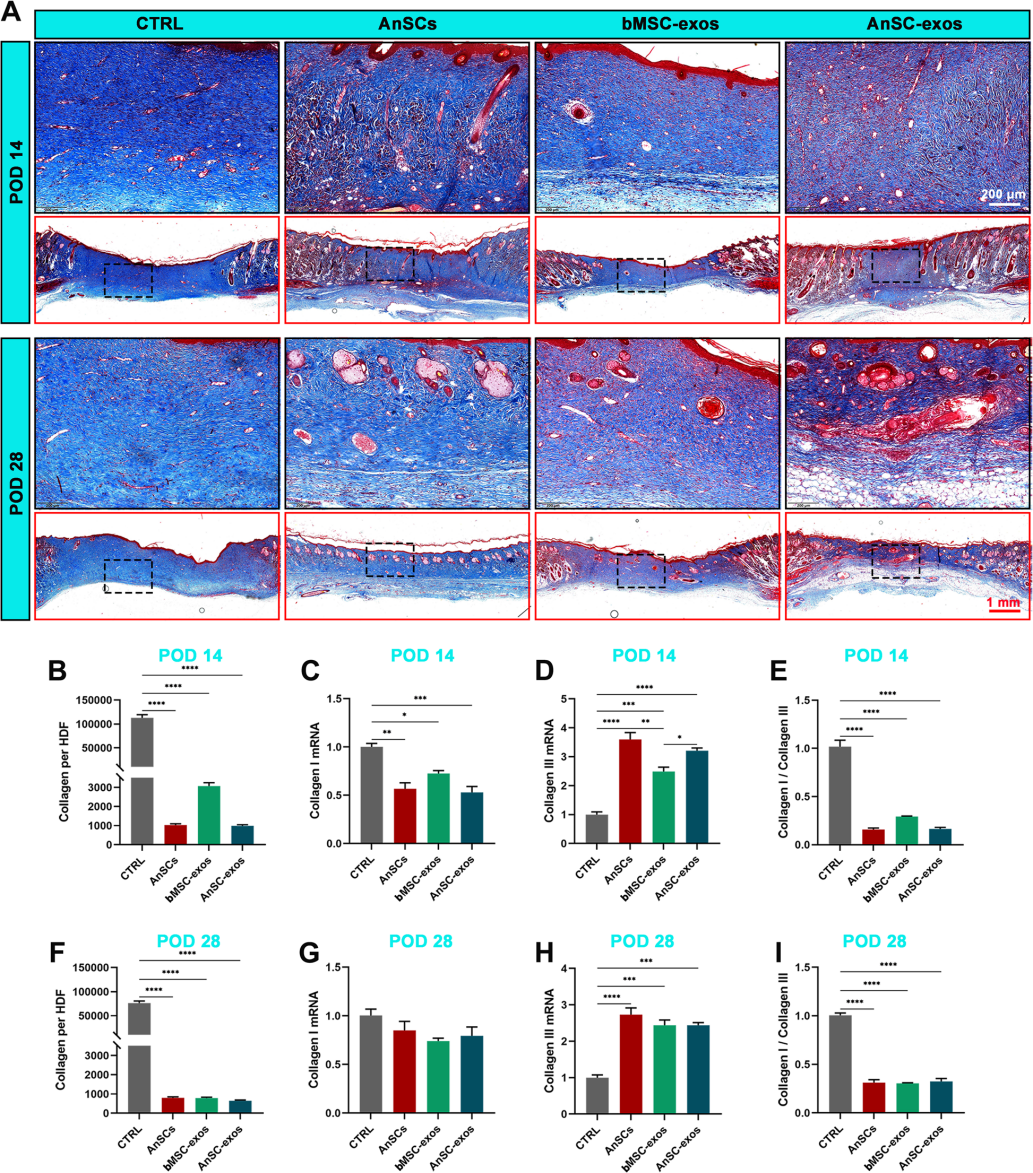
Effects of AnSC-exos on the structure and composition of collagen in model rats. **A** Histological sections of the healed skin stained with Masson. **B** Total collagen accounts in the healed skin on POD 14. **C**-**E** Relative mRNA levels of Collagen I and Collagen III, and their ratio on POD 14. **F** Total collagen counts in the healed skin on POD 28. **G**-**I** Relative mRNA levels of Collagen I and Collagen III, and their ratio on POD 28. Note: application of AnSC-exos reduced collagen abundance; and improved collagen structural composition, including higher expression of Collagen III genes (pro-regeneration) and lower expression of Collagen I genes (pro-scarring) in the wound healing. Mean ± SEM; *P < 0.05, **P < 0.01, ***P < 0.001, ****P < 0.0001; *n* = 3

The amount, type and composition of collagens were analyzed on POD14. Total collagens (blue area) were abundant in the healed skin of the CTRL group, whereas were significantly less in the AnSC-exos group than in the CTRL group (Fig. 4A, B; *P* < 0.0001). There was no significant difference in the amount of total collagen among the three treatment groups. Interestingly, the qRT-PCR results showed that expression of Collagen I mRNA in the CTRL group was higher than that in the treatment groups (Fig. 4C: AnSCs, bMSC-exos, AnSC-exos groups, *P *< 0.01, *P* < 0.05, *P* < 0.001 respectively), although there was no significant difference between the three treatment groups. In contrast, expression of Collagen III mRNA in the CTRL group was significantly lower than that in the AnSCs, bMSC-exos and AnSC-exos groups (Fig. 4D; *P* < 0.0001, *P* < 0.001 and *P* < 0.0001); expression level of Collagen III in the AnSC-exos group was higher than that in the bMSC-exos group (Fig. 4D; *P* < 0.05). There was no significant difference between the AnSC-exos group and the AnSCs group. The ratio of Collagen I mRNA to Collagen III mRNA of the CTRL group was higher than that of the treatment groups (Fig. 4E; *P* < 0.0001), but there was no significant difference among the three treatment groups. Similarly, compared to the CTRL group, AnSC-exos treatment significantly reduced the deposition of total collagens (Fig. 4A, F; *P* < 0.0001) and decreased expression of Collagen I mRNA, but increased expression of Collagen III mRNA in the healed tissue on POD28 (Fig. 4G-I; *P* < 0.01).

### AnSC-exos inhibited transition from fibroblast-to-myofibroblast (FMT)

**Fig. 5:**
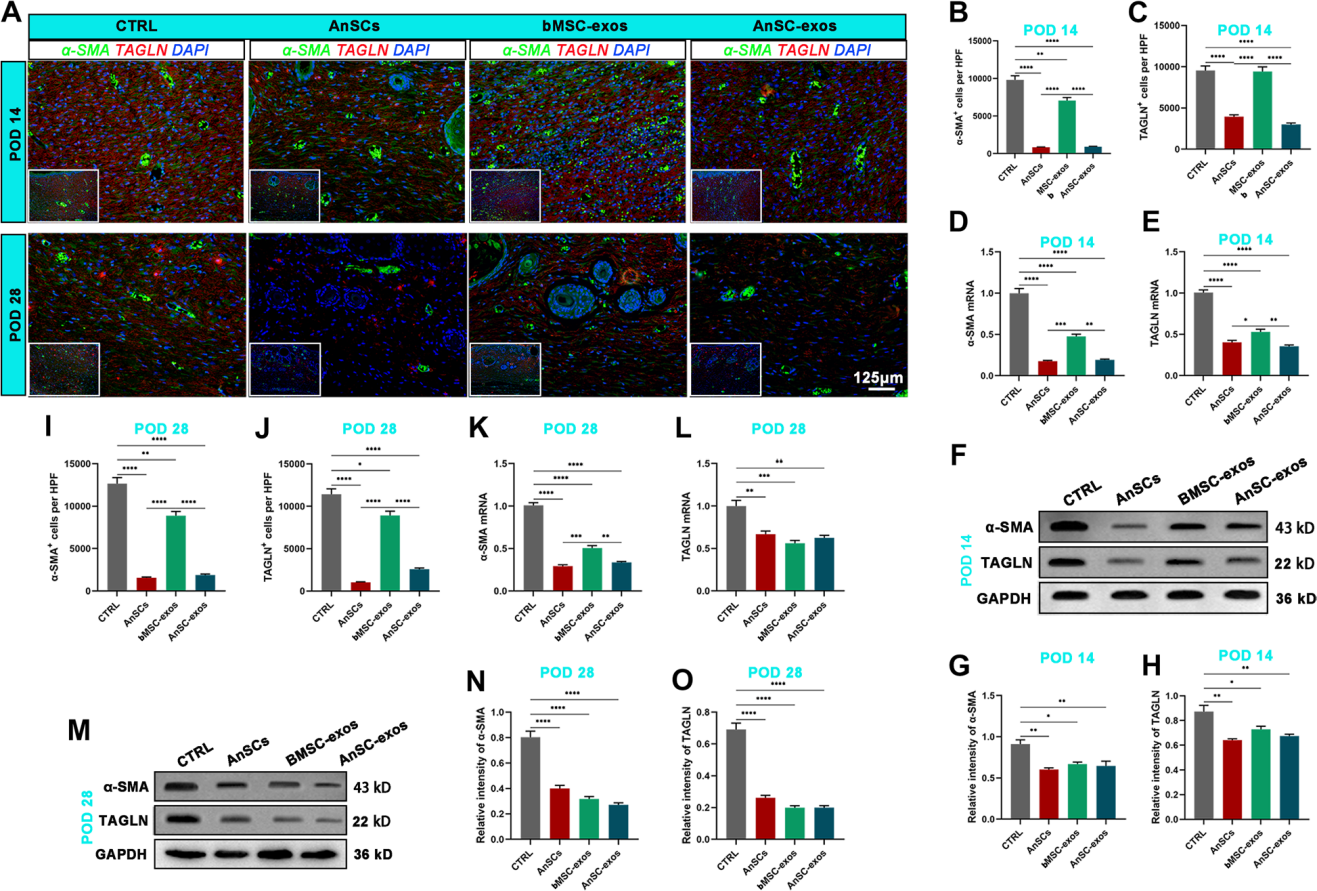
Effects of AnSC-exos on FMT in model rats. **A** Immunofluorescence stainings of α-SMA and TAGLN. **B**, **C** Numbers of α-SMA^+^ (except for blood vessel cells) and TAGLN^+^ cells on POD 14. **D**, **E** Relative mRNA levels of α-SMA and TAGLN on POD 14. **F**-**H** Western blot protein bands and relative intensities of α-SMA and TAGLN on POD 14. **I**, **J** Numbers of α-SMA^+^ (except for blood vessel cells) and TAGLN^+^ cells on POD 28. **K**, **L** Relative mRNA levels of α-SMA and TAGLN on POD 28. **M**-**O** Western blot protein bands and relative intensities of α-SMA and TAGLN on POD 28. Note: AnSC-exos down-regulated the expression levels of myofibroblast markers α-SMA and TAGLN, and this effect was comparable to that of AnSCs, but more potent than that of bMSC-exos. Mean ± SEM; *P < 0.05, **P < 0.01, ***P < 0.001, ****P < 0.0001; *n* = 3. α-SMA, α-smooth muscle actin; TAGLN, transgelin

FMT plays a critical role in the formation of fibrotic scar during wound healing. Therefore, investigation of FMT status of healing wounds treated with AnSC-exos in vivo and in TGF-β1-induced FMT in vitro could help understanding the underlying mechanism.α-SMA and Transgelin (TAGLN) are the two markers of myofibroblasts [10, 36]. We measured these two markers in the healed skins on POD14 and POD28 using IF. The results showed that the numbers of α-SMA^+^ cells and TAGLN^+^ cells in healed skin of the AnSC-exos group were significantly lower than those of the CTRL (*P* < 0.0001) and bMSC-exos (*P* < 0.0001) groups (Fig. 5A-C); and there were no significant differences between the AnSC group and the AnSC-exos group on POD 14. The changes in mRNA and protein expression levels of both α-SMA and TAGLN were further verified using qRT-PCR (Fig. 5D, E) and western blot (Fig. 5F-H), and similar trends to the IF results were obtained. Consistently, AnSC-exos treatment significantly decreased the expression levels of α-SMA and TAGLN in the healing tissue on POD28, evidenced via IF staining (Fig. 5A, I, J), qRT-PCR (Fig. 5K, L) and western blot analysis (Fig. 5M-O).

**Fig. 6:**
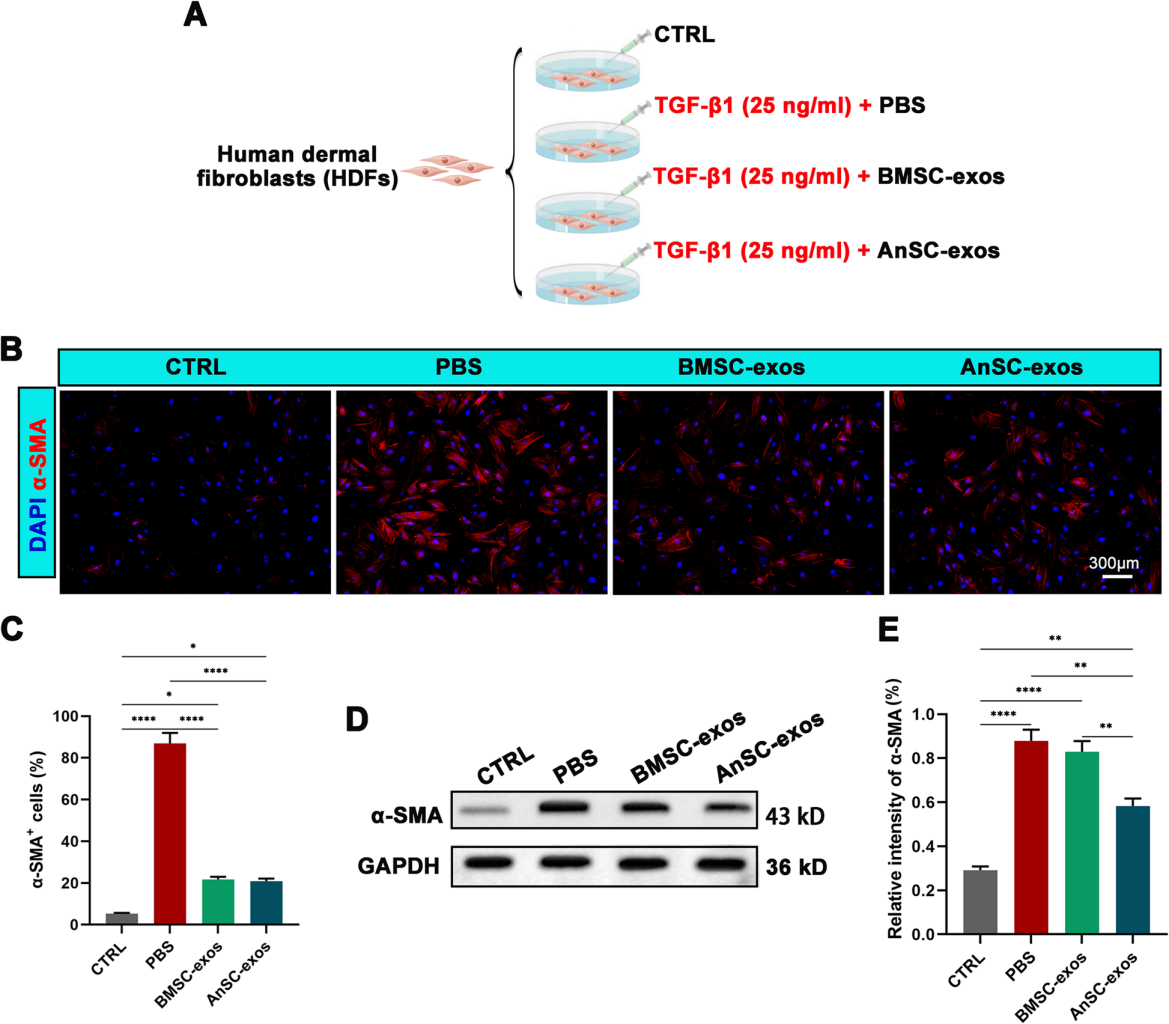
Effects of AnSC-exos on TGF-β1-induced FMT in vitro. **A** Schematic diagram of the experimental design. **B** Immunofluorescence staining of α-SMA. **C** Number of α-SMA^+^ cells. **D**, **E** Western blot protein bands of α-SMA and their relative intensities. Note: AnSC-exos reduced the expression level of α-SMA, and this effect was more potent than that of bMSC-exos. Mean ± SEM; **P < 0.01, ****P < 0.0001; *n* = 3. hDFs, human dermal fibroblasts

To further confirm the causal relationship between AnSC-exos application and FMT reduction, we carried out an in vitro study (Fig. 6A). The results showed that addition of TGF-β1 significantly increased markers of FMT, evidenced by the increase in number of α-SMA^+^ cells in the PBS group (TGF-β1 + PBS) in comparison to the CTRL group (intact) (Fig. 6B, C; *P* < 0.0001). Addition of AnSC-exos to the PBS group decreased the number of α-SMA^+^ cells (Fig. 6B, C; *P* < 0.0001). The expression level of α-SMA was further assessed using western blot, and a similar trend was found to the IF results (Fig. 6D, E). These results suggest that AnSC-exos can effectively inhibit TGF-β1-induced FMT. That is, AnSC-exos induced regenerative wound healing in vivo partially mediated via inhibiting FMT, which is achieved through down-regulation of the TGF-β1 pathway.

## Discussion

To the best of our knowledge, this is the first study to investigate the effects of exosomes derived from antler stem cells (AnSC-exos) on cutaneous wound healing. In the study, we found that topical application (injection) of AnSC-exos to full thickness wounds in rats effectively promoted the rate of healing and improved healing quality; and these effects were superior to those of bMSC-exos and comparable to the treatment with AnSCs. Further, in vivo and in vitro studies showed that promotion of regenerative wound healing by AnSC-exos was likely mediated through inhibition of FMT via down-regulation of TGF-β1 signaling transduction. Further characterization of AnSC-exos may help to identify more effective active components of the exosomes, potentially providing new options for regenerative wound healing in the clinical setting.

Wound healing is the process of restoration of cutaneous structure and function [1]. Healing generally leads to a fibrotic scar even under optimal conditions [2]. Therefore, understanding how to deflect wound repair from scar formation to a regenerative outcome remains the challenge for researchers in the field. In this respect, the lack of an appropriate animal model of scar-free wound healing under natural conditions has hindered the progress. However, large wounds (up to 10 cm in diameter or even more in some cases) on top of the deer pedicles (permanent bony protuberances from which antlers cast and regenerate) heal rapidly (within two weeks) and leave almost no visible scar [3–5]. This system, therefore, offers a rare opportunity to seek understanding scar-less wound healing in mammals.

In this respect, we firstly sought to understand the cell types involved in this type of regenerative healing; we have found that it is the closely-adjacent AnSCs, resident in the distal pedicle periosteum (PP) or the earliest PP-derived antler blastema, that provide the pedicle wound skin with the ability to achieve regenerative healing; thus this is achieved in a paracrine manner [37]. Secondly, we have shown that AnSC-induced regenerative wound healing is not skin type-specific through transplantation of AnSC tissue elsewhere to induce ectopic antlers; in so doing, somatic skin (facial) wounds, which were caused by casting of these ectopic hard antler, also achieved regenerative wound healing [38]. Thirdly, we demonstrated that AnSC-induced regenerative wound healing is not species-specific via injection of AnSCs to wounded rats [17, 39]. Interestingly, we also found that the topical application of AnSC-CM accelerated the closure rate and enhanced the quality of healing of full-thickness wounds in rats [29]. This work has laid the foundation for the isolation of effective agents that promote perfect wound healing.

Exosomes (membrane-bound vesicles with a diameter of 30 to 150 nm [30, 40]) are one of the main secreted products of MSCs. These exosomes carry various proteins, mRNAs and microRNAs that can modulate the phenotype of recipient cells and play an essential role in cutaneous wound healing [14, 15, 33, 34]. Compared with MSCs themselves, exosomes are simple to store and transport, and less risk of tumor generation and immune rejection. Therefore, in the present study, we isolated and purified AnSC-exos from the AnSC-CM and evaluated their effects on healing of full-thickness skin wounds in a rat model. As expected, AnSC-exos significantly decreased scar area and increased regeneration of cutaneous appendages, although in the present study, we have no way to pinpoint whether this scar reduction also contains contraction component, or purely achieved via tissue regeneration. The effects of AnSC-exos were more potent than bMSC-exos, and comparable to the AnSC treatment. Therefore, AnSC-exos may have potential to be developed as a novel cell-free therapeutic for scarless wound healing. In addition, our previous studies have shown that AnSCs had more potent cloning and proliferation potential compared to other MSCs, and can be readily passaged for over 50 generations, which means that AnSCs have the ability to produce more exosomes [22].

It is well established that excessive accumulation of collagens and wound contraction are the two main factors that cause scarring [3–5, 10, 41]. During the process of wound healing, some dermal fibroblasts transdifferentiate into myofibroblasts (FMT) [10, 12]. Myofibroblasts are not only the main cell type for collagen production, but also responsible for wound contraction [42]. Continued FMT and wound contraction causes formation of abundant thickened collagen bundles [43]. Consequently, we hypothesized that interruption of the process of FMT would help deflect wound healing from scar formation to regenerative restoration. Intriguingly, in this study, topic application of AnSC-exos on full-thickness rat wounds significantly reduced the indicators of FMT, indicating that the effects of AnSC-exos on regenerative wound healing are likely achieved through inhibition of FMT. It is known that exosomes derived from certain types of stem cells, such as epidermal stem cells [14], umbilical cord blood stem cells [34], and amniotic fluid stem cells [33], have also been reported to have the ability of inhibition of FMT. Given that the above MSC-derived exosomes all have the ability of inhibiting FMT, identifying their common functional components may pave the way for the identification of substances of inhibiting scar formation.

In addition to the deposition rate of collagen contributed by FMT, the type of collagen is another critical factor that affects wound healing. It is reported that the most striking feature is the persistence of high ratio of Collagen III over Collagen I (3:1) in scarless-wound healing in the fetus; whereas, this ratio is 1:3 in the adult (scarring) healed wounds [44]. Shamik Mascharak et al. [7] reported that higher levels of Collagen III yield smaller, reticular structures with more cross-linking than higher level of Collagen I, which contributes towards scarless wound healing. The present study found that the ratio of Collagen I/Collagen III in the healed wound tissue in the AnSCs or AnSC-exos treatment group was higher than that of the control group.

At this stage, the underlying molecular mechanism of the inhibition of FMT by AnSC-exos is speculative. TGF-β signaling is reported to play a critical role in FMT [45, 46]. During wound healing, TGF-β1 binds to its receptor, and the resultant complex phosphorylates Smads; the latter then activates the expression of downstream target genes including α-SMA, which leads to FMT. Therefore, either down-regulation of TGF-β1 expression or inhibition of Smad phosphorylation would impair FMT. Indeed, in the present study, application of AnSC-exos significantly down-regulated expression of TGF-β1, evidenced by qRT-PCR results in vivo (Fig. S1), and hence inhibition of FMT by the exosomes is likely due to substances that target the TGF-β signaling pathway.

In the present study, we found that AnSC-exos down-regulated expression of α-SMA (a marker of myofibroblasts) both in vivo and in vitro, and were more potent than bMSC-exos. It is known that the “cargoes” carried by exosomes are crucial to their function and that miRNAs are reported to be some of the most important substances [30]. For example, Luo et al. [47] reported that bMSC-exos inhibited expression of TGF-β receptor 1 due to let-7a-5p, the most abundant miRNA in bMSC-exos. In an unpublished work, we have identified the 10 most abundant miRNA species in the AnSC-exos as let-7b, let-7a, miR-21, let-7c, let-7g, let-7e, miR-423-5p, miR-100, let-7f, miR-184. In this respect, a number of miRNAs have been reported in MSC-exos, including those that target TGF-β1, TGF-β2, TGF-β receptor 1, TGF-β receptor 2 and Smad2, all of which have been reported to play roles in the inhibition of FMT in fibrotic diseases [14, 15, 32–34]. In fact, most of the miRNAs identified in the AnSC-exos are reported to target the TGF-β signaling pathway. Hence, we propose that these miRNAs account for at least part of the potent FMT inhibitory effect of AnSC-exos. Besides miRNAs, protein components in the AnSC-exos may also play a role in inhibition of the TGF-β signaling pathway. In our recent study [28], we found that protein factors in the AnSC-exos inhibited the expression of TGF-β1 in an osteoarthritis model.

**Fig. 7:**
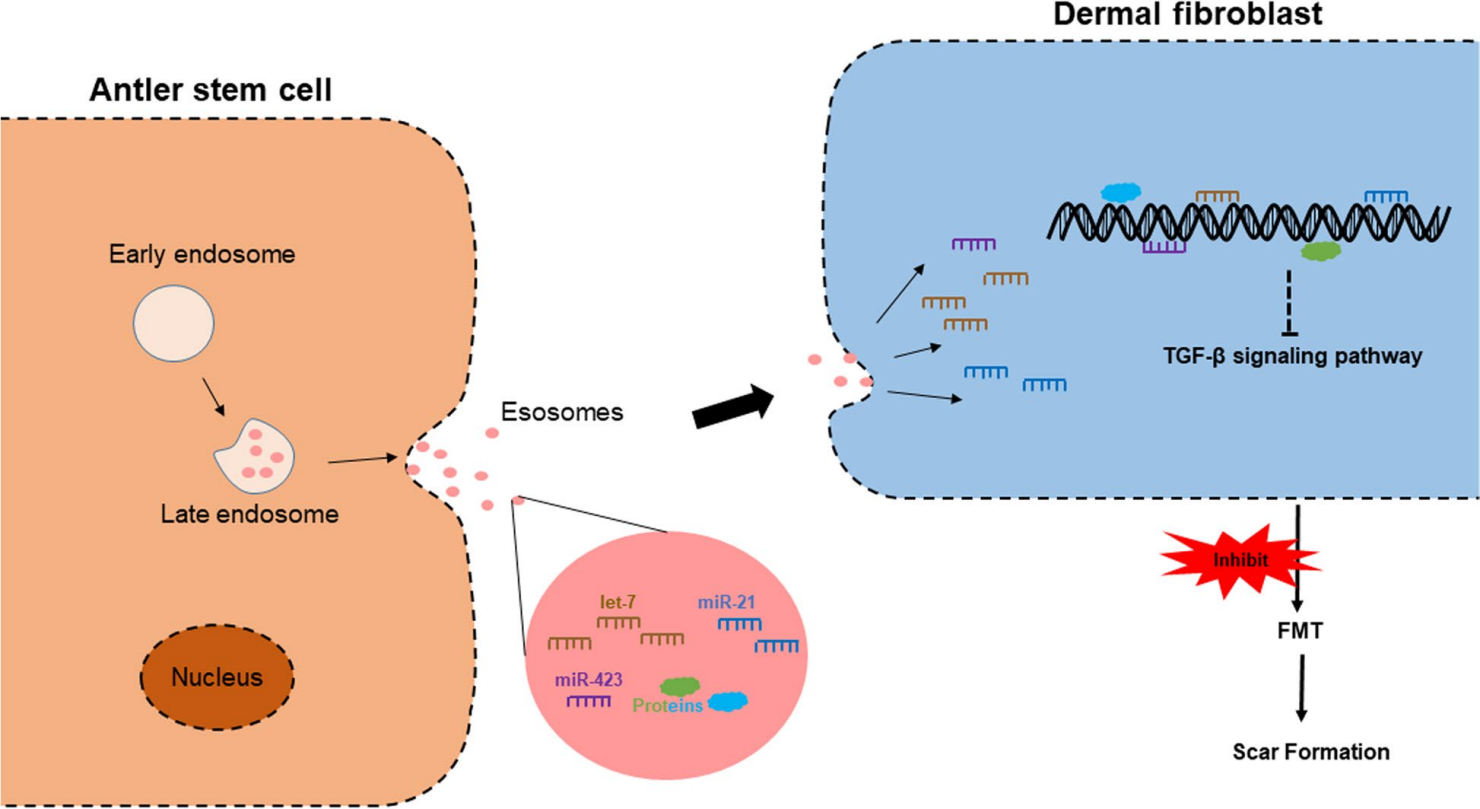
AnSC-exos promoted regenerative wound healing via FMT inhibition. We infer that it is the “cargos” of AnSC-exos including miRNAs (let-7, miR-21, miR-423, etc.) and protein factors that inhibit FMT via downregulating the TGF-β signaling pathway

Overall, our current working hypothesis is that during wound healing, miRNAs (such as let-7a-5p) and proteins from the AnSC-exos down-regulate the TGF-β signaling pathway, and that this, in turn, impairs FMT, whereby causing a reduction in collagen accumulation and wound contraction, and that the outcome is the improved quality of wound healing (Fig. 7).

## Conclusions

The present study revealed that AnSC-exos effectively enhanced the rate of healing and improved the quality of healing of full thickness skin wounds in rats, including regeneration of the cutaneous appendages and formation of the basketweave-like pattern of collagens. These effects of AnSC-exos were likely achieved through inhibition of the excessive FMT. Application of AnSC-exos may represent a novel approach for enhancing the quality of wound healing in the clinical setting.

### Supplementary Information

**Additional file 1: Table S1.** Antibodies. **Table S2.** Primers. **Fig. S1.** Effects of AnSC-exos on TGFβ1 expression in model rats. Relative mRNA levels of TGFβ1 on POD 14 and POD 28 via qRT-PCR. Mean ± SEM; *P < 0.05, **P < 0.01; *n* = 3.

## Data Availability

All data generated or analysed during this study are included in this published article [and its supplementary information files].

## Abbreviations

α-SMA: α-smooth muscle actin
AnSCs: Antler stem cells
AnSC-CM: AnSC conditioned medium
AnSC-exos: AnSC-derived exosomes
bMSC-exos: Bone marrow mesenchymal stem cell-derived exosomes
CK14: Cytokeratin 14
CK19: Cytokeratin 19
CTRL: Control
FCM: Flow cytometry
HE: Haematoxylin and eosin
hDF: Human dermal fibroblasts
HPF: 20× high-power field
IF: Immunofluorescence
POD: Postoperative days
TAGLN: Transgelin
TGF-β1: Transforming growth factor-β1
qRT-PCR: Quantitative real-time polymerase chain reaction
MSC: Mesenchymal stem cell
