# Supplementary material for: Antler stem cell-derived exosomes promote regenerative wound healing via fibroblast-to-myofibroblast transition inhibition
Source: J Biol Eng. 2023 Nov 8;17:67. doi: 10.1186/s13036-023-00386-0 (PMC10633995; doi:10.1186/s13036-023-00386-0)
Supplement: Supplementary file 1 — Additional file 1: Table S1. Antibodies. Table S2. Primers. Fig. S1. Effects of AnSC-exos on TGFβ1 expression in model rats. Relative mRNA levels of TGFβ1 on POD 14 and POD 28 via qRT-PCR. Mean ± SEM; *P < 0.05, **P < 0.01; n = 3. [file 13036_2023_386_MOESM1_ESM.docx]

**Supplemental materials**

**Table S1 Antibodies**

| **Antibody** | **Company** | **Catalog number** | **Dilution** |
| --- | --- | --- | --- |
| CD9 | Beyotime, China | AF1192 | 1:2000 (WB) |
| CD34 | Beyotime, China | AF1387 | 1:500 (IF); 1:100 (FCM) |
| CD45 | Beyotime, China | AF7839 | 1:500 (IF); 1:100 (FCM) |
| CD63 | Beyotime, China | AF1471 | 1:2000 (WB) |
| CD73 | Bioss, China | bs4834R | 1:500 (IF); 1:100 (FCM) |
| CD90 | Bioss, China | BS0778R | 1:500 (IF); 1:100 (FCM) |
| CD105 | Bioss, China | BS0579R | 1:500 (IF); 1:100 (FCM) |
| CK14 | ProteinTech, China | 10143-1-AP | 1:500 (IF) |
| CK19 | ProteinTech, China | 10712-1-AP | 1:500 (IF) |
| Sox2 | Bioss, China | BS0523R | 1:500 (IF); 1:100 (FCM) |
| TAGLN | ProteinTech, China | 10493-1-AP | 1:500 (IF); 1:2000 (WB) |
| TSG101 | Beyotime, China | AF8259 | 1:2000 (WB) |
| α-SMA | ProteinTech, China | 67735-1-Ig | 1:500 (IF); 1:2000 (WB) |
| GAPDH | Bioss, China | BS0755R | 1:2000 (WB) |
| Goat anti-Ms IgG/Cy3 | ProteinTech, China | SA00009-1 | 1:500 (IF, FCM) |
| Goat anti-Rb IgG/Cy3 | ProteinTech, China | SA00009-2 | 1:500 (IF, FCM) |
| Goat anti-Ms IgG/AF488 | ProteinTech, China | SA00013-1 | 1:500 (IF, FCM) |
| Goat anti-Rb IgG/AF488 | ProteinTech, China | SA00013-2 | 1:500 (IF, FCM) |

**Table S2 Primers**

| **Gene** | **F-Primer** | **P-Primer** |
| --- | --- | --- |
| Col1α2 | GGTGCCCCTGGAGAGAAT | GGACCAGCAGACCCAATG |
| Col3α1 | GTCCACGAGGTGACAAAGGT | CATCTTTTCCAGGAGGTCCA |
| TAGLN | CGGCCTTTAAACCCCTCACC | GACTGCACTTCTCGGCTCAT |
| α-SMA | AGCCATGTACGTAGCCATCC | CTCTCAGCTGTGGTGGTGAA |
| GAPDH | TGCCCCCATGTTTGTGATG | TGTGGTCATGAGCCCTTCC |


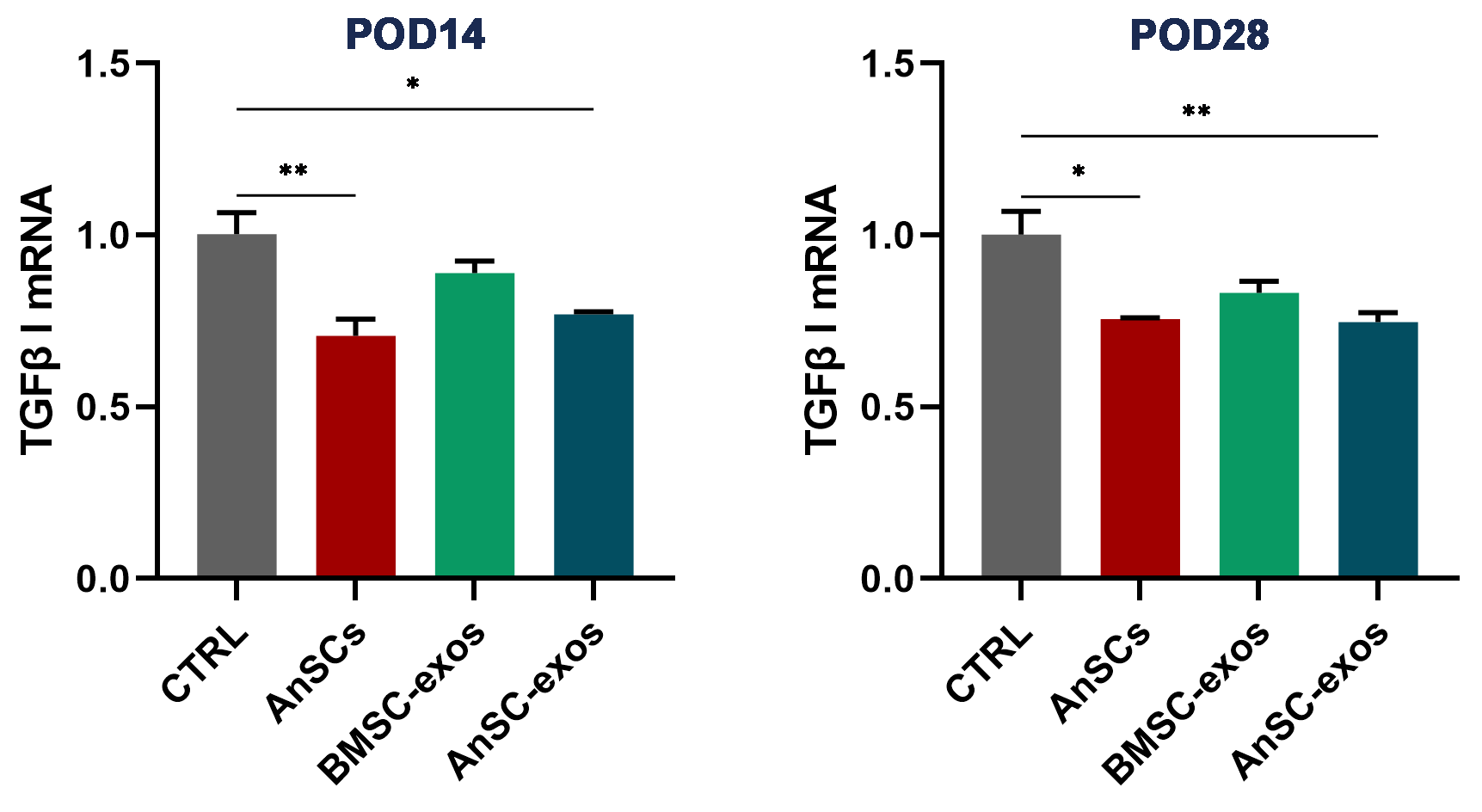


**Fig. S1** Effects of AnSC-exos on TGFβ1 expression in model rats. Relative mRNA levels of TGFβ1 on POD 14 and POD 28 via qRT-PCR. Mean ± SEM; *P<0.05, **P<0.01; n = 3.
